# Antifungal resistance and stewardship: a knowledge, attitudes and practices survey among pharmacy students at the University of Zambia; findings and implications

**DOI:** 10.1093/jacamr/dlad141

**Published:** 2023-12-21

**Authors:** Steward Mudenda, Scott Kaba Matafwali, Moses Mukosha, Victor Daka, Billy Chabalenge, Joseph Chizimu, Kaunda Yamba, Webrod Mufwambi, Patrick Banda, Patience Chisha, Florence Mulenga, McLawrence Phiri, Ruth Lindizyani Mfune, Maisa Kasanga, Massimo Sartelli, Zikria Saleem, Brian Godman

**Affiliations:** Department of Pharmacy, School of Health Sciences, University of Zambia, Lusaka PO Box 50110, Zambia; Antimicrobial Resistance Coordinating Committee, Zambia National Public Health Institute, Lusaka, Zambia; Clinical Research Department, Faculty of Infectious and Tropical Diseases, London School of Hygiene & Tropical Medicine, Keppel Street, London WC1E 7HT, UK; Department of Pharmacy, School of Health Sciences, University of Zambia, Lusaka PO Box 50110, Zambia; Department of Public Health, Michael Chilufya Sata School of Medicine, Copperbelt University, Ndola PO Box 71191, Zambia; Department of Medicines Control, Zambia Medicines Regulatory Authority, Lusaka PO Box 31890, Zambia; Antimicrobial Resistance Coordinating Committee, Zambia National Public Health Institute, Lusaka, Zambia; Antimicrobial Resistance Coordinating Committee, Zambia National Public Health Institute, Lusaka, Zambia; Department of Pharmacy, School of Health Sciences, University of Zambia, Lusaka PO Box 50110, Zambia; Department of Pharmacy, School of Health Sciences, University of Zambia, Lusaka PO Box 50110, Zambia; Department of Pharmacy, School of Health Sciences, University of Zambia, Lusaka PO Box 50110, Zambia; Conservation Department, World Wide Fund For Nature (WWF Zambia Country Office), Lusaka PO Box 50551, Zambia; Department of Pharmacy, Maina Soko Medical Center, Woodlands, Lusaka PO Box 320091, Zambia; Clinical Research Department, Faculty of Infectious and Tropical Diseases, London School of Hygiene & Tropical Medicine, Keppel Street, London WC1E 7HT, UK; Department of Epidemiology and Biostatistics, Zhengzhou University, College of Public Health, 100 Kexue Avenue, Zhengzhou, Henan 450001, China; Department of Surgery, Macerata Hospital, Macerata, Italy; Department of Pharmacy Practice, Faculty of Pharmacy, Bahauddin Zakariya University, Multan 60800, Pakistan; School of Pharmacy, Sefako Makgatho Health Sciences University, Ga-Rankuwa, Pretoria 0208, South Africa; Strathclyde Institute of Pharmacy and Biomedical Sciences, Strathclyde University, Glasgow G4 0RE, UK; Centre of Medical and Bio-Allied Health Sciences Research, Ajman University, Ajman 346, United Arab Emirates

## Abstract

**Introduction:**

Antifungal resistance (AFR) is a growing global public health concern. Little is currently known about knowledge, attitudes and practices regarding AFR and antifungal stewardship (AFS) in Zambia, and across the globe. To address this evidence gap, we conducted a study through a questionnaire design starting with pharmacy students as they include the next generation of healthcare professionals.

**Methods:**

A cross-sectional study among 412 pharmacy students from June 2023 to July 2023 using a structured questionnaire. Multivariable analysis was used to determine key factors of influence.

**Results:**

Of the 412 participants, 55.8% were female, with 81.6% aged between 18 and 25 years. Most students had good knowledge (85.9%) and positive attitudes (86.7%) but sub-optimal practices (65.8%) towards AFR and AFS. Overall, 30.2% of students accessed antifungals without a prescription. Male students were less likely to report a good knowledge of AFR (adjusted OR, AOR = 0.55, 95% CI: 0.31–0.98). Similarly, students residing in urban areas were less likely to report a positive attitude (AOR = 0.35, 95% CI: 0.13–0.91). Fourth-year students were also less likely to report good practices compared with second-year students (AOR = 0.48, 95% CI: 0.27–0.85).

**Conclusions:**

Good knowledge and positive attitudes must translate into good practices toward AFR and AFS going forward. Consequently, there is a need to provide educational interventions where students have low scores regarding AFR and AFS. In addition, there is a need to implement strategies to reduce inappropriate dispensing of antifungals, especially without a prescription, to reduce AFR in Zambia.

## Introduction

Antimicrobial resistance (AMR) is a term used to describe the ability of bacteria, fungi, parasites and viruses to overcome the lethal effects of antimicrobial agents.^1,2^ AMR poses challenges in treating infections resulting in increased morbidity, mortality and costs, with AMR increasingly seen as the next pandemic unless addressed.^3–7^ Within AMR, antifungal resistance (AFR) is seen as an increasing problem;^8–12^ however, the prevalence of AFR has been underestimated in the past.^13,14^ This misconception needs to be urgently addressed with a rise in fungal infections globally, particularly drug-resistant ones, further increasing morbidity and mortality rates from AMR.^9,14–17^ Currently, it is estimated that over 1.5 to 2 million people globally die each year from fungal infections, similar to the number of people dying each year from antibacterial resistance, with over a billion people currently affected by fungal diseases, which includes over 150 million people with severe fungal infections.^10,11,18,19^ The costs of treating patients with fungal diseases are also substantial, estimated at $6.7 billion–$7.2 billion annually in the USA alone in 2017 and 2018,^6,20^ with these costs expected to rise with increasing AFR.

Despite rising mortality and costs due to AFR, most emphasis, research and public health policies have primarily been focused on resistance to antibacterials and antivirals as opposed to antifungals.^11,21^ This skew has had unintended consequences of leaving AFR relatively neglected compared with antibiotic resistance (ABR), which is despite increasing concerns with AFR.^9,13,22^ This oversight has serious implications for the overall management of fungal infections, although this is starting to change with, for instance, the call for more research on AFR and the introduction of stewardship programmes to improve the utilization of antifungals.^23^ For instance, in Zambia, Nowbuth *et al*.,^24^ in their recent systematic review on published studies regarding the prevalence of AMR in Zambia, did not find any studies on AFR meeting their inclusion criteria. However, published studies exist on other resistant pathogens.^24^

Some drivers of AMR include the inappropriate prescribing of antimicrobials including subtherapeutic dosing,^8,13,25,26^ exacerbated by the inappropriate dispensing of antimicrobials without prescriptions.^27–29^ Alongside this, low awareness, inadequate knowledge and poor attitudes and practices concerning the use of antimicrobials have further increased AMR as a result of their irrational use.^29–32^

Antifungals are essential for effectively managing fungal infections.^33–35^ However, achieving their optimal use remains a challenge.^36^ The use of antifungal medicines has increased in recent years due to the increased burden of fungal infections, especially among immunocompromised individuals.^21,35,37^ Some commonly used antifungals include fluconazole, amphotericin B, miconazole, itraconazole, voriconazole, ketoconazole, posaconazole, isavuconazole, caspofungin, anidulafungin and micafungin, which are used to treat fungal infections including candidiasis, meningitis, histoplasmosis and oral thrush.^38–41^ However, there are concerns with their overuse and misuse, exacerbated by the switching of some antifungals from prescription medicines to over-the-counter medicines, exacerbating AFR.^38,42–44^ Alongside this, an appreciable number of patients using antifungal treatments do so improperly, often failing to complete the full course, leading to AFR.^45,46^ Furthermore, AFR fungi are typically resilient and transmissible human pathogens and thus potentiate the problem of AMR.^10,21,47^ AFR mainly occurs via efflux pump activation, drug target overexpression and amino acid substitution,^8,39,48,49^ with a number of studies now reporting AFR against commonly used antifungals.^8,50–57^ This situation is likely to worsen unless proactively addressed.^9^ This is a concern because AFR limits the number of effective antifungal therapies and causes treatment to be expensive, especially in low- and middle-income countries (LMICs).^11,13,23,58,59^

The ever-growing problem of AFR requires development and successful implementation of multiple strategies within countries, especially among LMICs.^5,60,61^ The first step in this process is to assess current awareness, knowledge and attitudes towards antifungals and AFR among key stakeholder groups in order to develop pertinent interventions to reduce identified problems. Secondly, to develop and implement pertinent strategies, which could include educational strategies as well as targeted antimicrobial stewardship programmes (ASPs).^60,62,63^ This is essential with sustainable antifungal stewardship programmes (AFSPs) needing to be developed and promoted globally to reduce AFR.^17,23,64–69^

As mentioned, the majority of studies assessing antimicrobial utilization and AMR in Zambia among patients, as well as ASPs, have typically focused on antibiotics, antivirals and anti-TB drugs, not antifungals.^24,70–76^ This needs to be urgently addressed given that Zambia has a significant population grappling with immune system-compromising diseases including HIV/AIDS, which presents a high risk of opportunistic diseases including fungal infections.^76–78^ Consequently, we sought to address this evidence gap by initially assessing the knowledge, attitudes and practices (KAP) of student pharmacists at the University of Zambia regarding AFR and AFS. This builds on similar studies regarding antibiotics and AMR among healthcare students in Zambia, as well as healthcare professionals (HCPs).^79–81^ The findings can subsequently be used to refine educational programmes in universities to help improve antifungal utilization in the future.

We started with student pharmacists as they are the future community pharmacists, with pharmacists globally increasingly dealing with infectious diseases and their treatment following COVID-19.^82–84^ In view of this, it is important that community pharmacists are fully conversant with key aspects of antifungals and AFR. We are aware in Zambia that up to 100% of pharmacies dispense antibiotics without a prescription.^85^ However, we are also aware that in Kenya and Namibia well-trained pharmacists give advice on the appropriate management of self-limiting infectious diseases without resorting to antimicrobials.^84,86–88^ In view of this, we believe the future pharmacists in Zambia should be able to provide appropriate advice to patients without unnecessarily exposing them to antifungals and AFR, especially those patients without medical prescriptions.

## Materials and methods

### Study design, population and site

A cross-sectional study was conducted among undergraduate pharmacy students at the University of Zambia between June 2023 and July 2023, according to the STROBE guidelines. To be eligible, participants had to be enrolled and registered as undergraduate pharmacy students at the University of Zambia, with active participation after obtaining informed and written consent.

The sample size was estimated using Taro Yamane’s formula,^89^ by employing a finite population of 601 undergraduate pharmacy students stratified as 196 second-year students, 158 third-year students, 133 fourth-year students and 92 fifth-year students. After adjusting for a 10% non-response, our final minimum required sample size was 265 students. The study population was classified into strata based on the year of study. All participants were identified using class registers and were randomly sampled using computer-generated random numbers.

### Data collection

Data were collected using an adapted questionnaire from two recent studies.^90,91^ Public health experts from the University of Zambia and the Copperbelt University subsequently reviewed the data collection tool for face and content validity. Hence, the questionnaire was pre-validated for simplicity, clarity, understandability, relevance and accuracy. The authors chose to adapt the data collection tools on AMR and AMS because there were no KAP studies that were done on AFR and AFS prior to the study. The questionnaire had four sections, namely: **Section A**: Sociodemographic characteristics of participants assessed using five questions; **Section B**: 10 questions on knowledge of AFR and AFS; **Section C**: 10 questions on attitude towards AFR and AFS; and **Section D**: 10 questions on practices regarding AFR and AFS. A pilot study was subsequently undertaken with 20 pharmacy students to improve the robustness of the questionnaire. Students who participated in the pilot study were excluded from the main study. The reliability of the questionnaire was determined using a Cronbach’s α value. Hence, with a Cronbach’s α value of 0.827 demonstrating an acceptable internal consistency, the final questionnaire was seen as reliable. The participants were recruited and provided with a self-administered questionnaire, which was submitted to the data collectors on completion. Data collection was undertaken by three data collectors (S.M., P.C. and W.M.) and lasted for 20 to 30 min per participant. A total of 420 questionnaires were administered to the identified participants.

### Study measures

**Figure 1.**
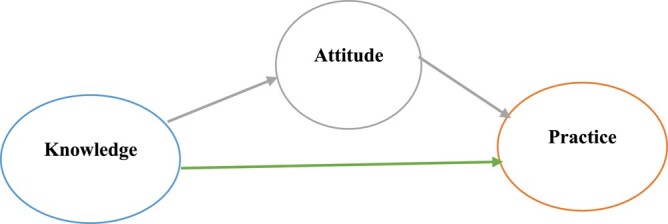
Knowledge directly (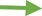) affecting outcome or mediated indirectly (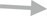) by attitude.

The main outcomes of this study were knowledge, attitudes and practices towards AFR and AFS (coded as good = 1, poor = 0). The KAP questions had three options (yes, no or neutral). Item scores were summed to obtain a composite score for each of the three options (Table S1, available as Supplementary data at *JAC-AMR* Online). The continuous scores were categorized to obtain binary variables for good knowledge, positive attitude and good practice using a cut-off value of 80%. We hypothesized that students with good knowledge and attitudes would have good practices towards AFR and AFS. In addition, the effect of knowledge on practice towards AFR and AFS will be mediated by students’ attitudes (Figure 1).

### Statistical analysis

We reported frequencies and percentages for categorical variables. The Pearson chi-squared test was used to compare scores of KAP among the students. KAP scores were calculated by adding correct responses to obtain a composite score. The scores were categorized into a binary variable using a cut-off value of 80% coded as ≥80% = 1 (‘good knowledge, attitude and practice’) and <80% = 0 (‘poor knowledge, attitude and practice’). Separate logistic regression models were used to calculate crude and adjusted ORs (AORs) with respective 95% CIs. All three multivariable models used significant variables at 20% from the univariable analysis. An investigator-led stepwise regression technique was used to drop off variables with high *P* values sequentially until a parsimonious model was built. Interactions were assessed between the final model’s significant variables, and none reached any statistical significance.

We further conducted generalized structural equation modelling to assess the interrelationships between variables and their mechanisms of association. We calculated direct, indirect and total effects to examine how knowledge affected practice towards AFR and AFS, part of which could occur through attitude. The effect of knowledge on practice towards AFR and AFS while controlling for attitude is called the direct effect. On the other hand, the indirect effect occurs because knowledge affects the attitude, which in turn affects the practice towards antifungal resistance. Ultimately, the direct and indirect effects form the total effects on the outcome (practice towards AFR and AFS). All models were independently adjusted for year of study. Bootstraps (50 replications) were used to compute standard errors for effects estimates. All statistical analyses were performed in STATA (version 17; StataCorp LP), and the significance level was set at α less than 5%.

### Ethics

This study was approved by the University of Zambia Health Sciences Research Ethics Committee (UNZAHSREC), approval number 2022112301176. Participants provided written informed consent after being informed of the purpose of this study. Participation in this study was voluntary and strictly for those who provided consent.

## Results

### Characteristics of study participants

Of the 420 questionnaires distributed, 412 were completed and returned, resulting in a response rate of 98%. The majority of the participants were female (55.8%), aged between 18 and 25 years (81.6%), unmarried (91.8%) and resided in urban areas (80.6%) (Table 1).

**Table 1.** Sociodemographic characteristics of participants (*N* = 412)

Variable	Category	*n* (%)
Age (years)	18–25	336 (81.6)
	26–33	50 (12.1)
	>33	26 (6.3)
Sex	Female	230 (55.8)
	Male	182 (44.2)
Residence	Rural/peri-urban	80 (19.4)
	Urban	332 (80.6)
Marital status	Unmarried	378 (91.8)
	Married	34 (8.3)
Year of study	Second	126 (30.6)
	Third	145 (35.2)
	Fourth	85 (20.6)
	Fifth	56 (13.6)

### KAP towards antifungals and AFR

Most students had good knowledge (85.9%), attitudes (86.7%) and practice (65.8%) towards AFR and AFS (Table 2). The highest proportion of participants who had good practice was among the third-year students (71.7%). There was no evidence though of an association between the year of study and scores for knowledge and attitudes.

**Table 2.** Students’ KAP according to the year of study

	Total (*N*= 412)	Year of study				*P* value
		Second (*N* = 126)	Third (*N*= 145)	Fourth (*N* = 85)	Fifth (*N* = 56)	
Knowledge, *n* (%)						
* *Poor	58 (14.1)	19(15.1)	21 (14.5)	11(12.9)	7(12.5)	0.954
* *Good	354 (85.9)	107(84.9)	124(85.5)	74(87.1)	49 (87.5)	
Attitude, *n* (%)						
* *Poor	55(13.4)	14(11.1)	17(11.7)	16(18.8)	8(14.3)	0.373
* *Good	357(86.7)	112(88.9)	128(88.3)	69(81.2)	48(85.7)	
Practice, *n* (%)						
* *Poor	141(34.2)	40(31.8)	41(28.3)	39(45.9)	21(37.5)	0.046
* *Good	271(65.8)	86(68.3)	104(71.7)	46(54.1)	35(62.5)	

This study found that 97.3% of the participants knew the definition of AFR, 93.7% could give examples of antifungals, 93.9% knew that misuse of antifungals contributes to AFR, and 89.3% knew that AFR can lead to prolonged illnesses and higher mortality. The lowest score among students concerned over-the-counter antifungal medicines not leading to AFR (Table 3).

**Table 3.** Students’ responses to the KAP statements

Domain	Yes, *n* (%)	No, *n* (%)	Don’t know/neutral, *n* (%)
Knowledge statements			
* *Antifungal resistance is a phenomenon where fungi become less responsive to antifungal medications.	401 (97.3)	4 (1.0)	7 (1.7)
* *Fluconazole, amphotericin B, and itraconazole are examples of common antifungal medications.	386 (93.7)	7 (1.7)	19 (4.6)
* *Misuse or overuse of antifungal medications contributes to the development of antifungal resistance.	387 (93.9)	9 (2.2)	16 (3.9)
* *Antifungal resistance could lead to prolonged illnesses and higher mortality rates.	368 (89.3)	18 (4.4)	26 (6.3)
* *Only bacterial infections can develop resistance; fungal infections cannot.	9 (2.2)	381(92.5)	22 (5.3)
* *It’s important to complete the full course of prescribed antifungal treatment, even if symptoms improve earlier.	403 (97.8)	4 (1.0)	5 (1.2)
* *Antifungal stewardship programs aim to improve the use of these drugs.	388 (94.2)	8 (1.9)	16 (3.9)
* *Over-the-counter antifungal medications cannot lead to antifungal resistance.	49 (11.9)	321 (77.9)	42 (10.2)
* *Patient adherence to the prescribed antifungal regimen is crucial for effective treatment.	375 (91.0)	12 (2.9)	25 (6.07)
* *Regular diagnostics are not necessary when prescribing antifungal treatment.	339 (82.3)	37 (9.0)	36 (8.7)
Attitude statements			
* *Antifungal resistance is a significant public health concern.	371 (90.1)	12 (2.9)	29 (7.0)
* *The current training and education about the proper use of antifungals and antimicrobial resistance are sufficient.	132 (32.0)	211 (51.2)	69 (16.8)
* *It is okay to prescribe antifungal medication even without a confirmed fungal infection.	15 (3.6)	387 (93.9)	10 (2.4)
* *All healthcare students should participate in antifungal stewardship programs.	379 (92.0)	1 (0.2)	32 (7.8)
* *Patient non-compliance to antifungal medicines contributes to the occurrence of antifungal resistance.	367 (89.1)	22 (5.3)	23 (5.6)
* *Overuse or misuse of antifungal medications in healthcare practices is a public health concern.	370 (89.8)	17 (4.1)	25 (6.1)
* *The proper use of antifungal medicines is a critical part of effective patient care.	396 (96.1)	5 (1.2)	11 (2.7)
* *It’s necessary to discuss antifungal resistance and its implications with patients.	405 (98.3)	2 (0.5)	5 (1.2)
* *I believe that more research is needed in the field of antifungal resistance.	399 (96.8)	4 (1.0)	9 (2.2)
* *Preventive measures, such as infection control and prophylaxis are important in managing antifungal resistance.	373 (90.5)	6 (1.5)	33 (8.0)
Practice statements			
* *I bought antifungal medicines without a prescription.	124 (30.2)	256 (62.1)	32 (7.8)
* *When my family/friend is sick, I recommend buying antifungals.	62 (15.1)	303(73.5)	47 (11.4)
* *I use antifungals because of advice from friends and family.	56 (13.6)	323 (78.4)	33 (8.0)
* *I use antifungals when I have a urinary tract infection.	95 (23.1)	277 (67.2)	40 (9.7)
* *I use antifungal medicines when I have a cold.	12 (2.9)	384 (93.2)	16 (3.9)
* *I seek additional education or training on antifungal medications and resistance.	334 (81.1)	39 (9.5)	39 (9.5)
* *Prescribing physicians and students are the only professionals who need to understand antifungal stewardship.	29 (7.0)	358 (86.9)	25 (6.1)
* *I participate in antifungal stewardship and awareness programs.	151 (36.7)	198 (48.1)	63 (15.3)
* *I keep myself updated about the latest research and guidelines regarding antifungal medications and antimicrobial resistance.	177 (43.0)	146 (35.4)	89 (21.6)
* *Formal teaching on the proper usage of antifungals among healthcare students is an intervention that may minimize the phenomena of antifungal resistance.	391 (94.9)	13 (3.2)	8 (1.9)

Most of the participating pharmacy students (90.1%) knew that AFR is a significant public health concern; however, the majority (51.2%) felt that the current training on antifungals and AFR in the university was not sufficient. Most students (89.8%) were also aware that the misuse and overuse of antifungals contributed to AFR, and 90.5% believed that infection prevention and control measures could help address AFR (Table 3).

This study also found that the prevalence of accessing antifungal medicines without a prescription was 30.2%. Additionally, 73.5% of participants did not recommend antifungals to their families or friends, did not use antifungals for UTIs, did not use antifungals when they had a cold, and did not participate in any AFSPs. Finally, 81.1% reported that they sought additional education or training on antifungals and AFR (Table 3).

### Factors associated with KAP towards AFR and AFS

Table 4 shows the results from the multivariable analysis. Male participants were less likely to report good knowledge of AFR and AFS than female participants (AOR = 0.55, 95% CI: 0.31–0.98). Similarly, fourth-year students versus second-year students (AOR = 0.44, 95% CI: 0.20–0.98) and students residing in urban areas versus rural/peri-urban (AOR = 0.35, 95% CI: 0.13–0.91) were less likely to report a positive attitude.

**Table 4.** Predictors of good KAP among Bachelor of Pharmacy students

Variable	Knowledge		Attitude		Practice	
	AOR (95% CI)	*P* value	AOR (95% CI)	*P* value	AOR (95% CI)	*P* value
Year of study						
* *Second	Ref		Ref		Ref	0.646
* *Third	1.07 (0.54–2.10)	0.844	0.88 (0.41–1.88)	0.749	1.13 (0.67–1.91)	**0.013**
* *Fourth	1.09 (0.48–2.44)	0.841	0.44 (0.20–0.98)	**0.043**	0.48 (0.27–0.85)	0.22
* *Fifth	1.17 (0.46–2.99)	0.74	0.60 (0.23–1.56)	0.295	0.66 (0.33–1.29)	
Sex						
* *Female	Ref					
* *Male	0.55 (0.31–0.98)	**0.042**				
Marital status						
* *Unmarried			Ref		Ref	
* *Married			3.33 (0.75–14.74)	0.113	2.75 (1.13–6.68)	**0.026**
Residence						
* *Rural/peri-urban			Ref			
* *Urban			0.35 (0.13–0.91)	**0.032**		

AOR, adjusted odds ratio; 95%CI, 95% confidence intervals. Boldface indicates statistical significance at 5%. Good knowledge, attitude and practice were scores of 80% or above.

Furthermore, fourth-year students were less likely to report good practices than second-year students (AOR = 0.48, 95% CI: 0.27–0.85). On the other hand, married students were more likely to report good practices than unmarried students (AOR = 2.75, 95% CI: 1.13–6.68).

### Mediation analysis: effect of knowledge on the practice towards AFR and AFS

Mediation analysis was performed to examine if attitude is a mechanism through which knowledge could affect the practice towards AFR and AFS (Table 5). Good knowledge was significantly associated with good practice, both directly and indirectly, through attitude. The total effect of good knowledge and attitude on good practices was 1.47-fold.

**Table 5.** Mediation analysis of the influence of knowledge on the practice towards AFR and AFS

	Mediator (attitude)	
Effects	OR (95% CI)	*P* value
Total	1.47 (0.82–2.12)	<0.001
Indirect	0.18 (0.01–0.36)	0.042
Direct	1.27 (0.57–1.97)	<0.001

The model was adjusted for year of study.

## Discussion

To the best of our knowledge, this is the first study to assess the KAP of undergraduate pharmacy students regarding AFR and AFS in Zambia. Approximately 86% of participating students reported good knowledge and attitude towards AFR and AFS, with approximately 66% reporting good practice. Male students were less likely to report good knowledge of AFR and AFS than female students. Similarly, fourth-year students versus second years, and students residing in urban areas, were less likely to report positive attitudes. Furthermore, fourth-year students compared with second-year students were less likely to report good practices. In mediation analysis, good knowledge was significantly associated with good practice, both directly and indirectly, through attitude.

Encouragingly, most pharmacy students had a strong understanding of AFR and AFS, similar to previous studies that had assessed students’ general knowledge of AMR and AMS across countries.^80,91–94^ However, our findings were better than those seen in other studies involving students.^95,96^ The good knowledge reported in our study may be because pharmacy students in Zambia are exposed to information about antifungals, antibiotics, antivirals and antiprotozoal drugs during training. Reassuringly as well, most pharmacy students knew the term AFR, examples of antifungals, factors that promote AFR, and AFS as a strategy for combating this public health issue. These findings corroborate similar studies where students knew the definition of AMR and predisposing factors as well as AMS/ASPs as ways forward to combat this public health problem.^97,98^ Interestingly, fourth-year students in our study were less likely to report good practices than second-year students, which contrasts with a study in Ghana that found that the level of AMR and AMS knowledge correlated with the year of study.^91^ Additionally, female students were more likely to have good knowledge of AFR and AFS than male students. This could be because female individuals tend to seek medical help and visit healthcare facilities where they are likely to receive information about antifungal medicines.

The present study found that most students had positive attitudes towards AFR and AFS, similar to previous studies on AMR in Zambia.^79,80^ However, our study found positive attitudes compared with the negative attitudes towards AMR and ABR among students in China.^32^ Despite most students having positive attitudes towards AFR and AFS in our study, 51.2% felt that the training they received on antifungals, AFR and AFS was insufficient. However, this is similar to a study in Colombia where most students felt the information they received on AMR and AMS during training was insufficient.^99^ Consequently, this calls for improved AMR and AMS information in undergraduate curricula in Zambia and beyond as well as integrating AFR into the curricula. We will continue to monitor this in the future.

The present study found slightly lower scores in practices of students regarding AFR and AFS compared with the scores in knowledge and attitudes, which also needs addressing when updating the curricula. Intriguingly, we found that 30.2% of the students had purchased antifungals without a prescription. This is only important if antifungals were inappropriate for the infection, with countries typically making antifungals available over the counter. We are aware of the appreciable misuse and overuse of antifungals, which needs to be avoided to reduce AFR. Our findings also revealed that very few students participated in AFS and awareness programmes. Additionally, very few students updated themselves about the latest research and guidelines regarding antifungal medications and AMS. These behaviours contributed to the low scores in practice recorded among the study participants. Community pharmacists and their assistants can play a key role here. Consequently, it is important that student pharmacists participate in AFS and awareness programmes during their training, which was not the case in our study. In addition, students pharmacists must take part in AFSPs to improve the future use of antifungals to reduce AFR. Alongside this, the students must update themselves on the latest research and guidelines to improve the care of patients, which is not happening currently. One surprising finding of our study was that fourth-year students reported lower rates of good practices compared with their second-year counterparts. This is because fourth-year students, having had more exposure to clinical settings, may feel overconfident and more inclined to self-medicate, leading to poor practices for themselves and patients post-qualification. Studies from Ethiopia and other countries also found that students in higher years of study tend to self-medicate because they learn more practical-oriented courses that increase their understanding of diseases and use of medicines.^100–103^ The observed variations suggest that educational strategies, and their impact on students’ practices, can differ by region and institution; consequently, any updated educational input needs to be targeted to the specific student body in question. Future research should explore the underlying reasons for this discrepancy to inform the development of more effective educational programmes.

Additionally, organizations such as the Pharmaceutical Society of Zambia (PSZ) should introduce short courses and continuing professional development (CPD) programmes that focus on AFS and the role of AFS. The Ministry of Health and the Zambia National Public Health Institute (ZNPHI) should also champion the promotion of research and awareness campaigns on AFR and support AFSPs.^22^

We are aware that there are limitations in our study. Firstly, the study was only conducted at a single university in Zambia. This implies that the findings of our study may not be generalized to all the universities in Zambia. Secondly, we employed a quantitative, cross-sectional design, which may constrain the depth of information gathered. The approach we used in our study may affect the depth of findings as participants are not allowed to give their detailed opinion on a subject matter. Despite these limitations, since our study is the first to highlight students’ KAP concerning AFR and AFSPs in Zambia, we believe the findings can serve as an impetus for researchers, health authorities and policymakers to integrate AFR into programmes to address AFR in Zambia and beyond. Further, we recommend multicentre studies on AFR and AFS among students in all universities in Zambia. Further, future studies should be conducted to explore the KAP of healthcare professionals on AFR and AFS.

### Conclusions

Overall, most students possessed good knowledge and attitudes towards AFR and AFS, which is encouraging since AFR and AFSPs are a poorly researched and under-researched field across Africa including Zambia. This underscores the need for targeted educational interventions in areas where students scored poorly such as insufficient training on AFR, not participating in AFS, not being up to date with information on AFR and AFS, and addressing access to antifungals without prescriptions.

## Supplementary Material

dlad141_Supplementary_Data

## Supplementary data

Table S1 is available as Supplementary data at *JAC-AMR* Online.
