# Supplementary material for: Antifungal resistance and stewardship: a knowledge, attitudes and practices survey among pharmacy students at the University of Zambia; findings and implications
Source: JAC Antimicrob Resist. 2023 Dec 21;5(6):dlad141. doi: 10.1093/jacamr/dlad141 (PMC10733812; doi:10.1093/jacamr/dlad141)
Supplement: dlad141_Supplementary_Data [file dlad141_supplementary_data.docx]

**Table S1**

**Antifungal Resistance and Stewardship: A Knowledge, Attitudes, and Practices Survey among Pharmacy Students at the University of Zambia; Findings and Implications**

**PART A (Tick the answer): Sociodemographics of participants**

| Age | 1. 18-25 (b) 26-33 (c) Above 33 years |
| --- | --- |
| Gender | 1. Female (b) Male |
| Year of Study | 1. Second (b) Third (c) Fourth (d) Fifth |
| Marital status | 1. Single (b) Married (c) Divorced (d) Widowed |
| Residency | 1. Urban (b) Rural (c) Rural-Urban |

**PART B (Tick the answer): Knowledge, Attitudes, and Practices questions**

| **#** | **QUESTIONS** | **Yes** | **No** | **Neutral** |
| --- | --- | --- | --- | --- |
| 1 | Antifungal Resistance is a phenomenon where fungi become less responsive to antifungal medications |  |  |  |
| 2 | Fluconazole, Amphotericin B, and Itraconazole are examples of common antifungal medications |  |  |  |
| 3 | Misuse or overuse of antifungal medications contributes to the development of antifungal resistance |  |  |  |
| 4 | Antifungal resistance could lead to prolonged illnesses and higher mortality rates. |  |  |  |
| 5 | Only bacterial infections can develop resistance, fungal infections cannot |  |  |  |
| 6 | It's important to complete the full course of prescribed antifungal treatment, even if symptoms improve earlier. |  |  |  |
| 7 | Antifungal stewardship programs aim to improve the use of these drugs. |  |  |  |
| 8 | Over-the-counter antifungal medications cannot lead to antifungal resistance. |  |  |  |
| 9 | Patient adherence to the prescribed antifungal regimen is crucial for effective treatment. |  |  |  |
| 10 | Regular diagnostics are not necessary when prescribing antifungal treatment. |  |  |  |
| 11 | Antifungal resistance is a significant public health concern |  |  |  |
| 12 | The current training and education about the proper use of antifungals and antimicrobial resistance are sufficient |  |  |  |
| **#** | **QUESTIONS** | **Yes** | **No** | **Neutral** |
| 13 | It is okay to prescribe antifungal medication even without a confirmed fungal infection. |  |  |  |
| 14 | All healthcare students should participate in antifungal stewardship programs. |  |  |  |
| 15 | Patient non-compliance to antifungal medicines contributes to the occurrence of antifungal resistance |  |  |  |
| 16 | Overuse or misuse of antifungal medications in healthcare practices is a public health concern. |  |  |  |
| 17 | The proper use of antifungal medicines is a critical part of effective patient care. |  |  |  |
| 18 | It's necessary to discuss antifungal resistance and its implications with patients. |  |  |  |
| 19 | I believe that more research is needed in the field of antifungal resistance. |  |  |  |
| 20 | Preventive measures, such as infection control and prophylaxis are important in managing antifungal resistance. |  |  |  |
| 21 | I bought antifungal medicines without a prescription. |  |  |  |
| 22 | When my family/friend is sick, I recommend buying antifungals. |  |  |  |
| 23 | I use antifungals because of advice from friends and family. |  |  |  |
| 24 | I use antifungals when I have a urinary tract infection. |  |  |  |
| 25 | I use antifungal medicines when I have a cold |  |  |  |
| 26 | I seek additional education or training on antifungal medications and resistance. |  |  |  |
| 27 | Prescribing physicians and students are the only professionals who need to understand antifungal stewardship. |  |  |  |
| 28 | I participate in antifungal stewardship and awareness programs. |  |  |  |
| 29 | I keep myself updated about the latest research and guidelines regarding antifungal medications and antimicrobial resistance. |  |  |  |
| 30 | Formal teaching on the proper usage of antifungals among healthcare students is an intervention that may minimize the phenomena of antifungal resistance. |  |  |  |

**Thank you for your participation**
